# Notes for Contributors

**DOI:** 10.1155/S1463924681000217

**Published:** 1981


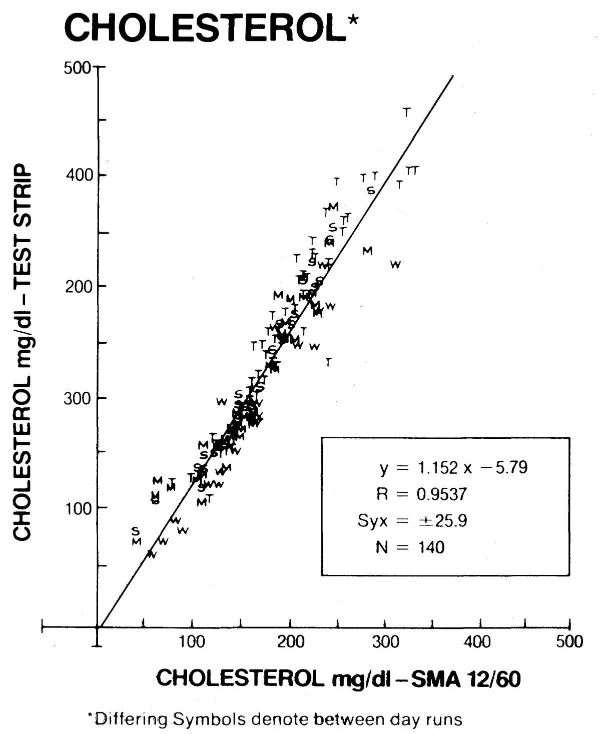

*Figure 10.*

using clinical specimens. Also presented in these figures, are the equations of the regression lines (y=mx +b) as well as the correlation coefficient (R), the number of points in the study (N), and the standard error of the estimate (Syx). The BUN test correlation data which are shown in Figure 8 indicate excellent correlation with the SMA 12/60 with slopes near 1 and a correlation coefficient exceeding 0.99. The LDH data are shown in Figure 9 and similar conclusions can be drawn. Finally, the cholesterol correlation data are shown in Figure 10 and also indicate excellent performance versus the reference method.

Correlations with reference methods are excellent and the C.V.'s obtained by means of these dry strips are comparable to other methodology currently considered acceptable by the medical community. Standard methods have also been compared against one another and the correlations curves were, in general, similar to those achieved with the strip tests. It is concluded, that this technology is highly promising. However, it must be emphasised that it is promising only if the systems are dry and self-contained. Since they offer no significant improvement in analytical capability, the dry systems' major advantages are convenience, simplicity and storageability. However, these are highly significant advantages and the authors are working to achieve a more complete battery of tests. In conclusion, this and related technology represent the imminent future of clinical chemical analyses.

REFERENCES

[1] Ross, J. and Frazer, M.D., (1979) *Am. J. Clin. Path.* 72, 265-273.

### Notes for Contributors

### Presentation of manuscripts

Manuscripts should be typed (double-spaced) on one side of the paper only and with generous margins. The title should be brief and informative avoiding the word "new" and its synonyms. The full list of authors with their affiliations and full address(es) should appear on the title page. On a separate sheet an abstract of no more than 150 words is required. This should succinctly describe the scope of the contribution and highlight significant findings or innovations. It should be written in a style which can easily be translated into French and German.

*The Concise Oxford Dictionary* and Fowler's *Modern English Usage* (both published by Oxford University Press) should be used as the standard for spelling and grammar. Abbreviations should be limited to those generally recognised, or where a frequently occuring term is abbreviated it should, in the first instance, be explained thus "flow injection analysis (FIA) ..." and the abbreviation used thereafter. Abbreviations, for standard measures and units should follow SI recommendations. There are various publications giving guidance on the use of SI units.

References should be indicated in the text by numerals following the author's name, i.e. Skeggs [6]. On a separate sheet of paper, list all references in numerical order thus:

[6] Skeggs, L.T., *American Journal of Clinical Pathology,* 1959, **28**, 311.

Note that journal titles are given in full. Where there is more than one author, the form Foreman et al. should be used in the text but all authors should be named in the list of references. When reference is made to a chapter in a book the reference should take the following form:

[7] Malmstadt, H.V. in "Topics in Automatic Chemistry" Ed. Stockwell P.B. and Foreman J.K. 1978 Horwood, Chichester, pp. 68-70.

Only work which has been published or has been accepted for publication should be cited. Avoid the citation of documents which are subject to restricted circulation, patent literature, unpublished work and personal communications. The latter can be mentioned in the text in parenthesis.

To illustrate a paper line diagrams are preferred to photographs. Photographs should only be used when they significantly add to the discussion. Diagrams, charts and graphs should be carefully drawn in black ink on stout card or heavy quality tracing paper. Most illustrations are reduced for publication; to allow for this originals should be between 16 and 36 cm wide (the depth must not exceed 50 cm). The lettering of diagrams should be sufficiently clear to withstand reduction. Except in the case of proper names, all lettering should be in lower case print. If photographs are used they must be supplied in the form of clear, unmounted, glossy, black and white prints. "Instant" photographs are not normally acceptable. All illustrations must be identified on the reverse showing the figure number and the author's name.

Each illustration should have a fully explanitory caption. Captions should be typed together on a separate sheet of paper; they must *not* be an inseparable part of the illustration.

